# IMMUnity Unveiled: A Translational NETwork for tackling PARKinson's Disease – IMMUPARKNET

**DOI:** 10.12688/openreseurope.17547.1

**Published:** 2024-06-19

**Authors:** Mitilda Gugu, Shubhra Acharya, Dogukan Pira, Simona Poletti, Alessia di Flora, Tamara Saksida, Vladimirs Pilipenko, Marina Romero-Ramos, Franca Marino, Laura Muñoz Delgado, Neda Nikolovski, Yasemin Gursoy Ozdemir, Dale Lawson, Cristoforo Comi, Inês Figueira

**Affiliations:** 1 Preclinical Department, Faculty of Technical Medical Sciences, University of Elbasan “Aleksandër Xhuvani”, Elbasan, Albania; 2 Cardiovascular Research Unit, Department of Precision Health, Luxembourg Institute of Health, Luxembourg, Luxembourg; 3 Luxembourg and Faculty of Science, Technology and Medicine, University of Luxembourg, Luxembourg, Luxembourg; 4 Faculty of Medicine, Izmir University of Economics, Izmir, Turkey; 5 Department of Translational Medicine, University of Eastern Piedmont, Novara, Italy; 6 Center for Research in Medical Pharmacology, School of Medicine, University of Insubria, Varese, Lombardy, Italy; 7 Institute for Biological Research "Siniša Stanković", National Institute of Republic of Serbia, University of Belgrade, Belgrade, Serbia; 8 Department of Pharmacology, Faculty of Medicine, University of Latvia, Riga, Riga, Latvia; 9 Danish Research Institute of Translational Neuroscience – DANDRITE & Department of Biomedicine, Aarhus University, Aarhus, Central Denmark Region, Denmark; 10 Unidad de Trastornos del Movimiento, Servicio de Neurología y Neurofisiología Clínica, Instituto de Biomedicina de Sevilla, Hospital Universitario Virgen del Rocío/CSIC, Universidad de Sevilla, Seville, Andalusia, Spain; 11 Department of Neurology, School of Medicine, Koç University, Istanbul, İstanbul, Turkey; 12 University of Eastern Piedmont, Novara, Italy; 13 S. Andrea Hospital, Vercelli, Italy; 14 NOVA Medica School | Faculdade de Ciências Médicas (NMS|FCM), Universidade Nova de Lisboa, Lisbon, Lisbon, Portugal

**Keywords:** Parkinson's disease, immune response, network

## Abstract

Parkinson’s disease (PD) affects more than one million people in the EU. It currently has no definitive cure, meaning that patients rely only on symptomatic treatments, which themselves are burdened by side effects. The need for advancements in both knowledge and available treatments is thus strongly felt by patients, caregivers, and health operators. This unmet need sparked the idea of orchestrating a collaborative effort via a common network – IMMUPARKNET (The role of IMMUnity in tackling PARKinson’s disease through a Translational NETwork).

The IMMUPARKNET COST Action focuses on challenges in PD and its related crosstalk with immune response. Although widely recognized, the role of immunity in the onset and development of PD is still unclear. The main goal of IMMUPARKNET is to fill this knowledge gap by establishing an innovative, interdisciplinary research network and fostering exchanges of expertise among specialists from different countries and institutions.

As we gather scientists and clinicians who study immunity in PD and related fields, IMMUPARKNET will establish the first nucleus of a multidisciplinary ecosystem that aims to harmonize efforts and approaches, both in research and clinical practice, to boost the development of ground-breaking treatments for PD. Through meetings, training schools, webinars, position papers, and review manuscripts, IMMUPARKNET will lead fruitful exchanges of know-how among experts in the field.

The IMMUPARKNET structure revolves around 5 working groups, with a total of 157 active members from 34 different countries. Of these active members, 58.5% are young researchers, while 67.5% come from Inclusiveness Target Countries (ITC - less research-intensive COST Members; https://www.cost.eu/about/members/).

IMMUPARKNET output will facilitate the improved sharing and development of research resources, straightening the road to novel treatments and identifying where existing ones can be repurposed, all, ultimately and hopefully, finding a cure for PD.

## Plain Language Summary

Parkinson’s disease (PD) affects more than one million people in the EU. It currently has no definitive cure, meaning that patients rely only on symptomatic treatments, which themselves are burdened by side effects. The need for advancements in both knowledge and available treatments is thus strongly felt by patients, caregivers, and health operators. This unmet need sparked the idea of orchestrating a collaborative effort via a common network – IMMUPARKNET (The role of IMMUnity in tackling PARKinson’s disease through a Translational NETwork).

Although widely recognized, the role of immunity in the onset and development of PD is still unclear. The main goal of IMMUPARKNET is to fill this knowledge gap by establishing an innovative, interdisciplinary research network and fostering exchanges of expertise among specialists from different countries and institutions.

## Disclaimer

The views expressed in this article are those of the authors. Publication in Open Research Europe does not imply endorsement of the European Commission.

## 1. Parkinson’s disease state-of-the-art and knowledge gaps

Parkinson’s disease (PD) is an age-related progressive neurodegenerative movement disorder that causes both motor and non-motor disability, significantly affecting patient quality of life. It affects more than 10 million people worldwide and is the second most common neurodegenerative disease after Alzheimer’s disease. According to the World Health Organization (WHO), disability-adjusted life years (DALYs) from PD increased by 81% and deaths by over 100% between 2000 and 2019 ( WHO). Due to the continuous aging of the population, these numbers are expected to double by the year 2030 ^1^. Although the disease is typically diagnosed when classic motor symptoms, such as bradykinesia, rigidity and/or tremor appear ^2^, a prodromal PD phase, characterized by a variety of non-motor symptoms, may precede the manifestation of motor symptoms by more than 10–15 years ^3^. Pathologically, PD occurs due to a progressive loss of dopaminergic neurons in the mesencephalic part of the brain, ‘ *substantia nigra pars compacta*’; the region responsible for dopamine production and motor-function maintenance. Neurodegeneration spreads further to other parts of the brain leading to the appearance of non-motor symptoms and disease progression ^4^. To date, neurodegeneration in PD is known to be irreversible, meaning that there is no cure. Moreover, currently available PD treatments rely on recovering dopamine levels in the brain, thereby providing only partial symptomatic relief to patients.

It is important to stress that PD is a multifactorial disorder. While the etiopathology of PD is still unknown, it is thought to result from the complex interplay between aging, genetic susceptibility, and exposure to environmental factors. As such, several interlinked mechanisms govern the degeneration of dopaminergic neurons, which include disease-causing genetic mutations ( *e.g., LRRK2, GBA, PINK1, PRKN*), and Lewy body (LB) formation ^5, 6^. The appearance of LBs is the histopathological hallmark of PD. LBs are abnormal aggregates of the protein α-synuclein (α-syn) that form toxic intracellular inclusions in the neurons. This accumulation of LBs is due to aggressive mutations, post-translational modifications, and perturbations in proteasomal degradation ^5^. Additionally, cell organelle dysfunction, such as in mitochondria, ER, and lysosomes, increases oxidative stress, synaptic dysfunction, neuroinflammation, and apoptosis, leading to dopamine dysregulation ^7^. Despite current knowledge of the above-mentioned mechanisms, the role of the immune system in PD remains elusive. The pathogenesis of PD is not only associated with neurons but also with the surrounding glial cells, which are required to maintain neuronal homeostasis ^8^. As such, both the innate and adaptive immune systems play a role in PD ^9^. Processes such as gliosis occur in the brain, in parallel with dopaminergic degeneration, causing the pro-inflammatory activation of the microglia and astrocytes ^10^. In the periphery, a proinflammatory immune response has been described as well as a reduction in circulating T lymphocytes in the blood of PD patients, compared to controls ^11^. Additionally, the breakdown of the blood-brain barrier (BBB) has been shown to cause T-cell infiltration from the periphery to the brain in mouse models and in postmortem brain samples ^12, 13^. In this regard, it can be stated that both central and peripheral immune responses and both innate and adaptive immunity are significantly involved in PD pathogenesis. However, there is still a need to understand the complex interplay between the periphery and central nervous system (CNS), and the role this plays in PD progression. An understanding of this crosstalk could add to our current grasp of the disease, as well as being useful in identifying reliable biomarkers and developing new disease-modifying therapies.

### 1.1 The social and economic burden of PD

There is an intrinsic relationship between health and economy, and understanding the social and economic impact of a disease is crucial for resource planning by healthcare systems and for raising social awareness of the disease.

**Figure 1.**
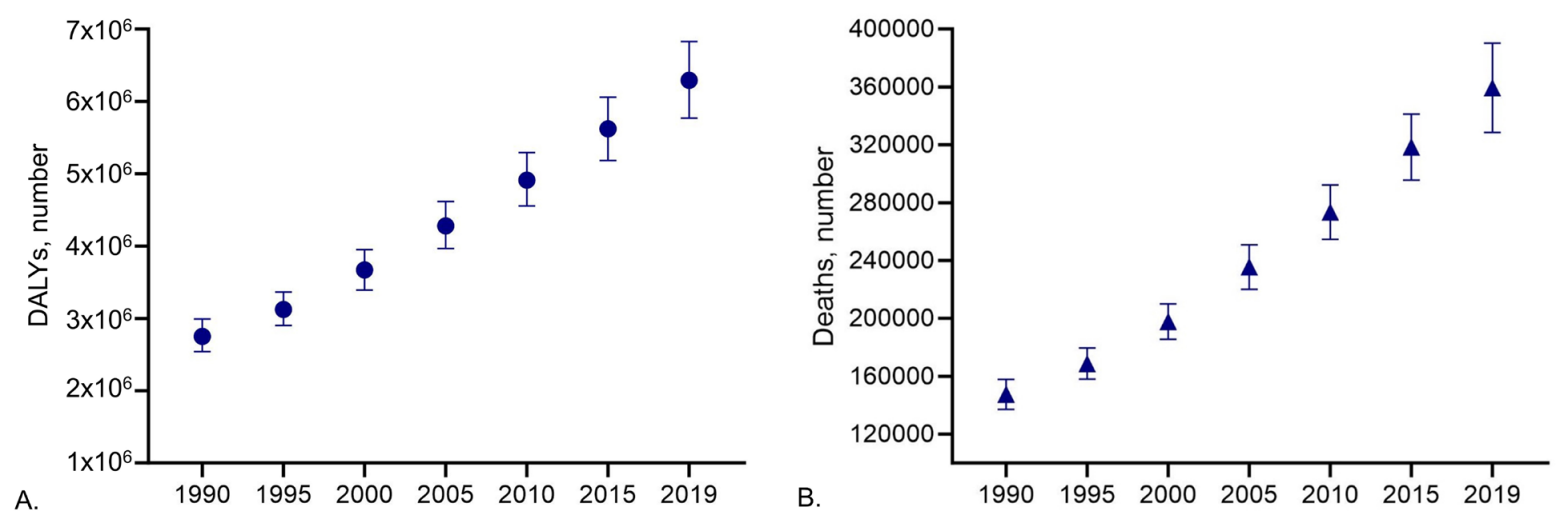
Global estimate of number of ( **A**) disability-adjusted life years (DALYs) and ( **B**) deaths of individuals living with PD from 1990 to 2019. Data obtained from GBD study ^16^.

In neurodegenerative diseases, such as PD, the burden of disease for society is often difficult to bear, whether it is financial, social, or emotional, and includes patient-related factors (disability, need for care, treatment, and early death) and environmental factors, such as social, economic, and family support. In terms of numbers, PD prevalence has doubled over the past 25 years. Global estimates in 2019 indicate that there were over 8.5 million individuals living with PD, with 5.8 million disability-adjusted life years (DALYs), an increase of 81% since 2000 ( Figure 1 A), and 329,000 deaths, an increase of over 100% since 2000 ( Figure 1 B) ^14^.

If we consider the increase in population life expectancy and the concomitant increase in the YLDs (Years Lived with Disease) for PD (i.e., circa 3 years), it is reasonable to assume that the socioeconomic burden of PD will likely worsen in the coming years.

The increasing prevalence of PD in individuals aged 60 and above is expected to lead to a substantial rise in associated costs, posing significant challenges to patients, families, caregivers and global health systems, particularly in low and middle-income countries (LMICs) with limited healthcare resources ^15^. The costs generated by a disease can be categorized as either direct costs or indirect costs. Direct costs are generated mainly by primary care, secondary care, and treatments, whereas indirect costs refer mainly to productivity losses caused by early patient retirement and decreased participation in the workforce by informal caregivers (family members and/or friends) and have a significant emotional and social impact that overcomes the economic aspects of the disease. Non-medical costs, for example the travel costs for obtaining care, must also be considered. A “ *cost of illness*” analysis provides the economic costs of an illness, injury and risk factors and includes all these types of costs.

Over the past 20 years, several studies have described the socio-economic consequences of PD. Although these studies have often covered all the main aspects of the social and economic burden of PD, the most recent one’s date back to 2016. If we consider European Union and COST Action countries, only a few are covered in these studies, suggesting that an extensive “cost of illness” study should be designed and conducted within COST countries in the near future ^17^.

The latest extensive study in Europe, conducted by the European Cooperative Network for Research, Diagnosis, and Therapy of Parkinson’s Disease (EuroPa) in six countries (Austria, Germany, Italy, Czech Republic, Portugal, and Russia), reveals significant variations in PD-related costs. Based on a 6-month survey of 486 patients and a societal perspective, total mean costs per patient ranged from EUR 2,620 (Russia) to EUR 9,820 (Austria) ^18^, with direct costs constituting 60–70% of the total. Western countries generally exhibited higher costs than Eastern nations. Another study comparing PD costs in the USA, Europe and Australia reported mean costs of EUR 17,064/patient/year in the USA, EUR 7,020 in Australia, and from EUR 649 to 9,544 in Asia, with values reflecting differences in healthcare system levels and capacities ^19^. The report published in Spain in 2013 by the Spanish Foundation for the Brain, not only presented similar findings on costs, but also investigated the social costs of PD, in particular the effects of the disease on the families ^20^.

Together with its heterogeneity and complex disease pathobiology, the lack of effective therapeutics and/or preventive approaches with which to tackle PD contributes to the tremendous socioeconomic burden it holds for society. Moreover, the emotional burden affects not only patients, but also their caregivers, and difficulties are increased by psychological and psychiatric disorders such as depression, dementia, and psychosis, which are manifestations of the advanced stages of the disease ^20^.

## 2. Parkinson’s disease and immune response: current challenges

**Figure 2.**
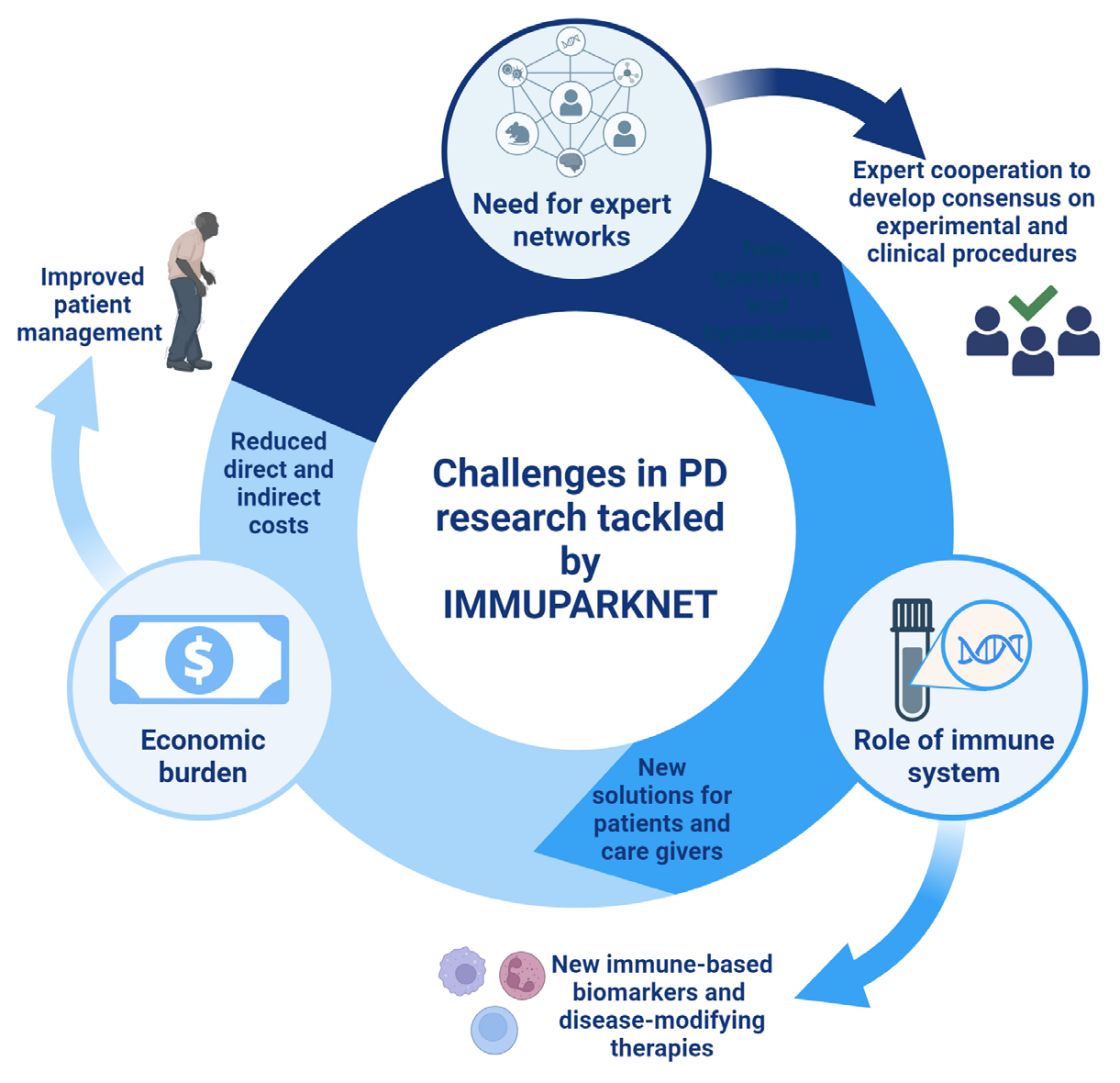
Current challenges in Parkinson’s disease (PD) researched addressed by IMMUPARKNET. Created in Biorender.com.

Despite promising advances and interesting future avenues in PD research, there are still several knowledge gaps and challenges that need to be addressed. Some of the challenges and proposed solutions are discussed below ( Figure 2).

### 2.1. Development of effective and standardized disease-modifying treatments for PD

The effectiveness of PD treatment is hampered by the limited "druggability" of the pathogenetic mechanisms found to date. This stems from various factors that impede effective treatment. One key challenge lies in the BBB, which restricts the passage of many medications into the brain, hindering their ability to target the specific areas affected by PD. Moreover, the late diagnosis of PD often results in treatments being initiated at advanced stages of cell degeneration, making it incredibly challenging to tackle the disease's progression effectively. This delay in diagnosis significantly limits the efficacy of available therapies. In this regard, research into the immune system's role in PD holds promise for developing disease-modifying therapies and immune-based biomarkers for improved diagnosis and treatment.

### 2.2. Launching initiatives to advance preclinical and clinical research

Findings on immunity in PD are fragmented and limited, preventing their translation into clinical practice. Limitations in current knowledge include heterogeneous preclinical models and methodologies, a lack of standardized clinical assessments, small patient samples and few longitudinal studies. To address these limitations, there is a need for consensus on experimental and clinical procedures, more dialogue between basic and clinical researchers, and multicenter studies.

### 2.3. Establishing sufficient collaboration between experts

Multidisciplinary cooperation between experts in PD, other neurodegenerative diseases and immunology is essential for advancing the state of the art in PD research. Currently, this cooperation is insufficient, which is a major obstacle to progress. Bridging the gaps between basic and clinical research, and between neurological and immunological sciences, is crucial to better understanding PD pathogenesis and developing effective therapies.

### 2.4. Lowering the expenses of treating patients with PD

The management of PD is costly and entails both direct and indirect costs. Direct costs include inpatient care and nursing homes, while indirect costs include lost productivity and carer burden. The cost estimates vary across countries, but PD costs billions of dollars each year in the US alone. The development of disease-modifying drugs that can slow or stop the progression of PD could benefit the lives of millions of people living with PD worldwide.

## 3. IMMUPARKNET as a solid network to fill the knowledge gap

IMMUPARKNET (COST Action CA21117), a four-year European Commission-funded COST action focused on studying the role of immunity in tackling PD, was launched on September 20, 2022, and will run through to the autumn of 2026, bringing together a network of 27 different countries. IMMUPARKNET is a unique initiative that aims, for the first time, to enhance the knowledge transfer between experts in the field of neurodegeneration and immunity to overcome the aforementioned challenges in PD research, leading to the development of innovative treatments for PD patients.

### 3.1. Paramount role of immune response in PD

**Figure 3.**
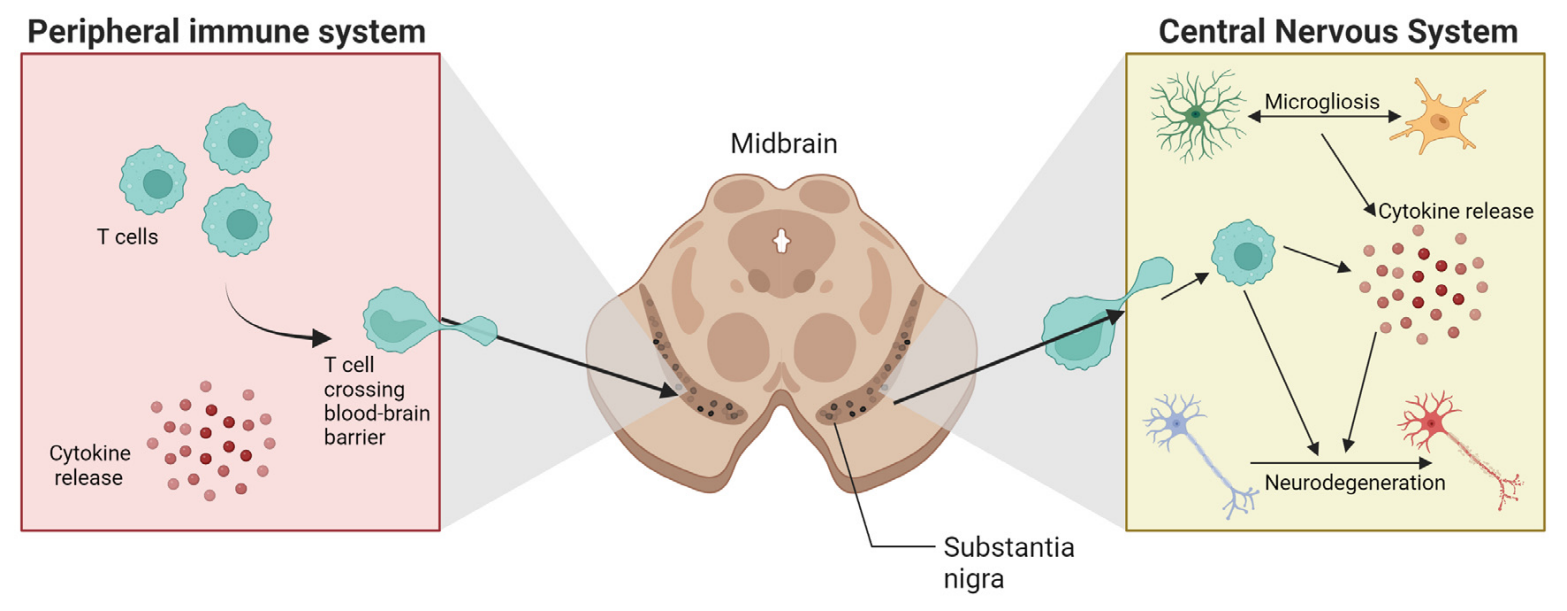
Crosstalk between peripheral and central nervous systems in PD development. Created in BioRender.com.

Neuroinflammation is increasingly recognized as a crucial factor in the development of various neurodegenerative disorders, such as PD. It was over 10 years ago, in 2013, that the work of Kannarkat and colleagues described the role of inflammation and the involvement of the immune system in PD, both in animal models and in human patients ^21^. Indeed, a discussion about the activation states of immune cells and their implications in PD susceptibility and therapeutic strategies has already been reported by Moehle and West ^22^. Additionally, researchers have emphasized the early and dynamic nature of immune-system responses in PD that involve both the central and peripheral immune systems ^9^ ( Figure 3). Indeed, the involvement of the immune system is not limited to the specific area in which the disease manifests, such as the *substantia nigra* in PD, but extends to changes in other areas of the CNS and the peripheral immune system. It is still unclear whether alterations observed in the peripheral immune system result from the process of cell degeneration in the CNS, or whether they act as primary agents in neurodegeneration. Schonhoff *et al*. have found that the immune system can be dysfunctional in PD and associated with pro-inflammatory responses, while Arlehamn *et al*. have discussed the involvement of the adaptive immune system, particularly T-cell responses, in PD and Alzheimer's disease ^23, 24^. Stone *et al*. have also emphasized the links between T-cell immunity and the degeneration of dopaminergic neurons in PD ^25^. Nevertheless, as the population ages, neurodegenerative disorders become more prevalent, and are often linked to changes in peripheral immunity ^26^. The ongoing debate revolves around whether aging itself contributes to the development of neurodegenerative diseases, or whether the aging process leads to the hyperactivation of immune and inflammatory pathways, thereby promoting neurodegeneration ^27^. Altogether, these findings indicate the importance of understanding the immune system's contribution to PD and suggest potential immunomodulatory therapies for either the prevention or treatment of the disease.

***As its mission, IMMUPARKNET COST Action will:***

**a)**  *help to share and compare models, protocols, and techniques among scientists by creating a network of excellence that brings together major groups working on immunity in PD in clinics and the lab, at a European and global level.* This collaborative approach will facilitate the exchange of knowledge, enabling researchers to take advantage of a wide range of methodologies and approaches to address the current challenges and problems in understanding the key immune dysregulations in PD, in terms of the cellular and molecular pathways involved.
**b)**  *enable the conduction of larger multicenter clinical studies by fostering collaboration among some of the most distinguished clinical centers.* This will lead to a more comprehensive and consolidated assessment of PD and its immunological aspects, improving the quality and generalizability of clinical research findings.
**c)**  *promote the development of novel research pathways to evaluate the pathogenetic mechanisms of PD in in-vivo and in-vitro models at the cellular and molecular levels, by bridging the gap between clinical and preclinical researchers.* By facilitating the crosstalk between clinical and laboratory-based investigators, novel research directions can be explored, enhancing our understanding of PD from multiple angles.
**d)**  *ensure that the scientific community remains attentive to the latest and most pressing issues related to immunity and PD, allowing for a more agile and dynamic response to new challenges and discoveries in this field.*
**e)**  *enhance scientific understanding while also helping to increase awareness among different stakeholders.* This includes policymakers, funding agencies and pharmaceutical companies who can play pivotal roles in supporting research, driving policy changes, and advancing treatments based on the latest scientific insights into the role of immunity in PD.

## 4. IMMUPARKNET organization and objectives

### 4.1. IMMUPARKNET structure

The IMMUPARKNET Cost Action is led by the Action Chair, Dr. Cristoforo Comi (Italy), and by the Action Vice-Chair, Dr. Yasemin Gursoy Ozdemir (Turkey).

**Figure 4.**
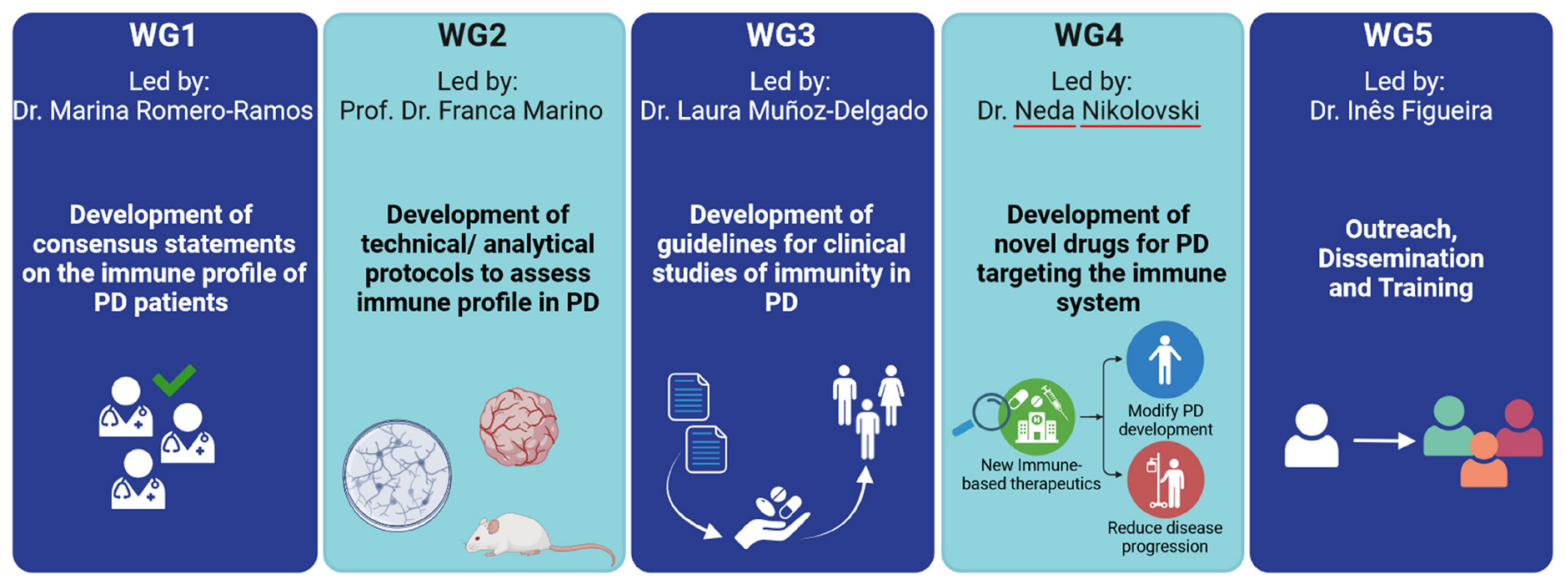
IMMUPARKNET working groups (WG) and main objectives. Created in Biorender.com.

IMMUPARKNET structure revolves around 5 interrelated working groups (WG), led by experts from different European countries. To date, IMMUPARKNET includes 157 active members from 34 different countries, including Azerbaijan. Of all these active members, 58.5% are young researchers, while 67.5% come from inclusiveness target countries. Moreover, circa 23% are clinicians and 77% are scientific researchers. The consortium will achieve its objectives through the coordination of these 5 WG ( Figure 4), which will each contribute in a distinct, but collaborative, manner: WG1 and WG2 will mainly concentrate on investigating the immune system's role in the development of PD; WG3 will develop guidelines for conducting clinical studies related to immunity in PD; WG4 will work on novel drug development targeting the immune system in PD; and, WG5 serves as a transversal working group to accomplish the capacity-building and dissemination objectives of the network.

The management activities of the Working Groups will be supported by continuous management structures and mechanisms to ensure the implementation of the Action according to the planned schedule and top-level management approaches, including the following bodies:

1. The Action Management Committee (AMC) made up of at least one representative from each COST Full Member. It is the principle decision-making body and is responsible for monitoring the implementation of the Action and overseeing financial resources.
2. The Scientific Committee (SC) consisting of the Chair, Vice-Chair, Grant-Holder Manager, Working Group Leaders, and other organizational roles, including the Science Communication Manager and Grant Awarding Coordinator. It makes proposals to the AMC, monitors the implementation of the Working Groups, ensures quality standards are met and disseminates Network activities.

### 4.2. IMMUPARKNET Working Groups (WG)

***4.2.1. WG1: Development of consensus statements on the immune profile of PD patients*.** Led by Dr. Marina Romero-Ramos (Denmark), WG1 aims to systematically collect and critically assess all existing preclinical and clinical information on the immune system in PD, as well as its connection to neuroinflammation and neurodegeneration. This includes examining relevant *in-vitro* and *in-vivo* models and identifying useful biomarkers for monitoring disease progression. Additionally, WG1 will focus on pinpointing potential drug targets within the immune system for creating disease-modifying PD treatments. The group aims to disseminate its findings to the scientific community through various communication channels, such as publications and meetings.

***4.2.2. WG2: Development of technical/analytical protocols to assess the immune profile in PD*.** Led by Prof. Franca Marino (Italy), WG2 aims to assess the latest advancements in the models, methodologies and techniques utilized to study immunity in PD patients and animal models of the disease. This includes defining and validating a set of "gold standards" to promote consistent research practices and disseminating accessible technical guidelines and protocols for PD via open-access platforms.

***4.2.3. WG 3: Development of guidelines for clinical studies of immunity in PD*.** The goal of WG3, led by Dr. Laura Muñoz-Delgado (Spain), is to establish the ideal blueprint for clinical study protocols that explore the role of immunity in PD, the development and progression of the disease, and the treatment response to anti-Parkinson’s drugs. A further objective is that of determining the optimal design for evaluating the therapeutic efficacy of immune-targeting drugs in PD.

***4.2.4. WG4: Development of novel anti-PD drugs that target the immune system*.** Led by Dr. Neda Nikolovski (Serbia), WG4 will strive to identify "druggable" immune-system targets that may potentially alter the progression and development of PD and will evaluate the treatments for immune-mediated disorders that are currently used to treat multiple sclerosis and other autoimmune conditions in order to find suitable candidates that can be repurposed as anti-Parkinson’s agents.

***4.2.5. WG5: Outreach, Dissemination and Training*.** The primary goals of WG5, led by Dr. Inês Figueira (Portugal), include ensuring the widespread dissemination of the latest knowledge generated by IMMUPARKNET to the scientific community, communicating the outcomes of the project’s actions to the general public, and fostering expertise development, with a particular focus on younger researchers and clinical practitioners.

### 4.3. IMMUPARKNET members

**Figure 5.**
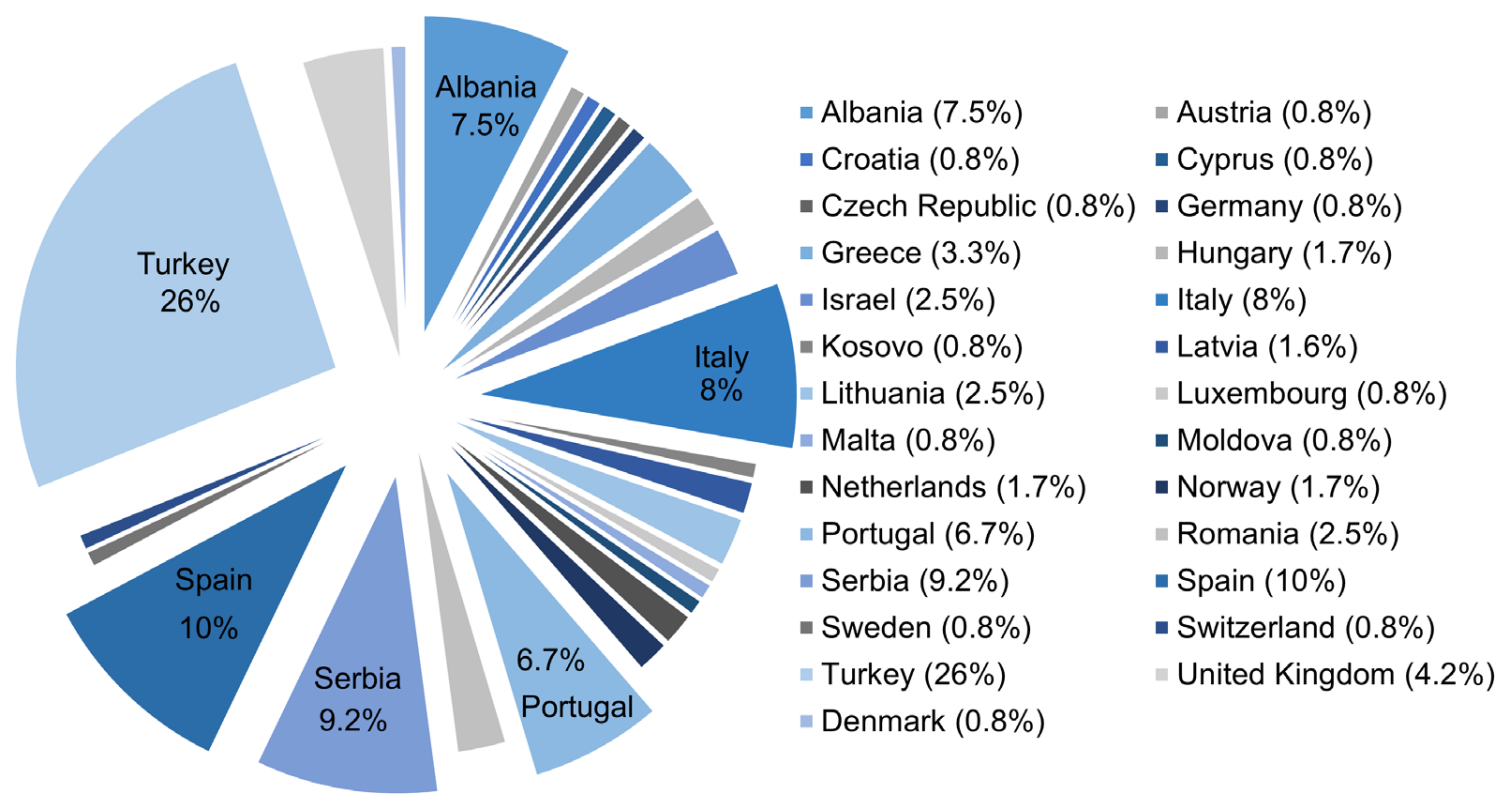
Percentage distribution of IMMUPARKNET Membership per country.

To date, IMMUPARKNET has 158 active members from 27 different countries. Turkey leads the count with 31 active members, followed by Spain (12), Serbia (11), Italy (10), Albania (9) and Portugal (8) ( Figure 5). Collectively, these countries account for 68% of the current active membership in IMMUPARKNET. Among the active members, 67% are females and 33% are males. 50% of the current members are young researchers under the age of 40 years. Additionally, an impressive 34% of the current membership comes from Inclusiveness Target Countries (ITC). These numbers indicate the diversity inherent in this COST Action, which can proudly call upon a broad spectrum of experience, perspectives and expertise that contribute to the inclusive nature of IMMUPARKNET.

**Figure 6.**
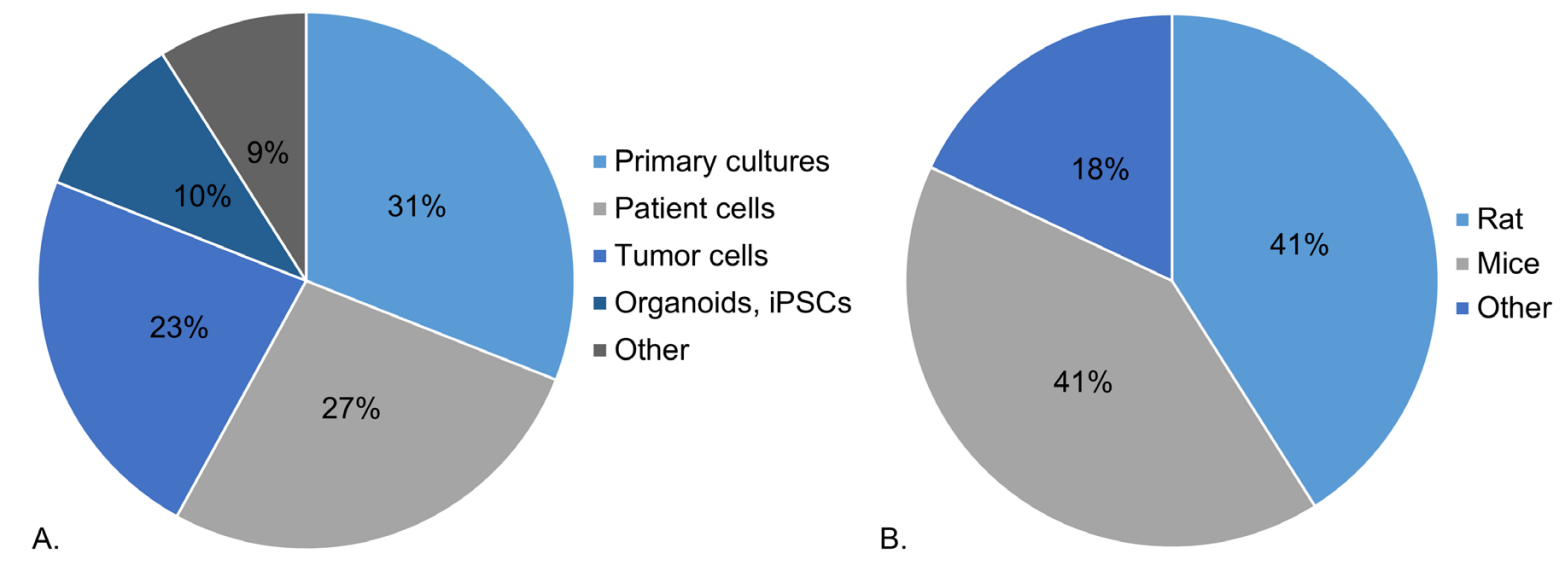
*In-vitro* and *in-vivo* models used by IMMUPARKNET members in their research ( **A**) Percentage distribution of different cell types, ( **B**) Percentage distribution of animal models.

***4.3.1. IMMUPARKNET member profiles*.** A questionnaire has been sent to all Action members and their responses can be found below. More than half of questioned IMMUPARKNET members (54%) work with cell lines in their research. As seen in Figure 6 A, approximately one third (31%) work with primary cultures, followed by patient cells (27%) and tumor cells (23%). The remaining types of cells used include organoids, induced pluripotent stem cells and other cell types, such as neural progenitor and buffy-coat-derived cells.

Moreover, more than half (55%) of Action’s members do not work with animal models in their research, while 31% work with animal models of PD. Most commonly, as seen in Figure 6 B, rats and mice are used to model PD, constituting 41% of PD model animals used by IMMUPARKNET members, followed by other models, such as Zebrafish and *Drosophila melanogaster* (18%). Importantly, more than half of IMMUPARKNET members (52%) have access to patient samples and/or data, yet only 26% of members generate immune profiles for PD patients.

## 5. Community engagement commitment by IMMUPARKNET

IMMUPARKNET utilizes a diverse range of approaches to enhance stakeholder engagement with, and the understanding and recognition of, the importance of immunity in PD. All members of IMMUPARKNET are invited to collaborate and actively take part in the following initiatives:

– Maintaining the IMMUPARKNET website ( https://immuparknet.eu), which presents Action aims, expected outcomes, public deliverables, events, blogs, etc.
– Organizing international conferences, whether that be the Action’s own or, potentially, held in collaboration with existing scientific annual events that are relevant to the disciplinary domains. This is done for the purposes of networking and the dissemination of project results.
– Organizing training events (seminars, workshops, webinars) for early career researchers and healthcare professionals.
– Publishing the scientific articles, consensus documents and guidelines generated by IMMUPARKNET Network in open-access international peer-reviewed journals, repositories, and websites.
– Identifying and connecting with other relevant projects, and inviting their representatives to join, or actively collaborate with, the Network.
– Writing newsletters and policy briefs to provide all stakeholders with news of the relevant achievements of the project in order to increase engagement.
– Engaging in dialogue with the general public and establishing an online community on the topic through social media accounts ( LinkedIn, Twitter).
– Preparing leaflets with IMMUPARKNET goals and activities for distribution among individuals and organizations at key locations/events (e.g., Parkinson’s Associations in different EU countries, international scientific and/or medical conferences, etc.).
– Using relevant educational materials, including videos and webinars, to disseminate network approaches and results to various non-specialist audiences, including patients and their organizations.
– Undertaking media outreach to disseminate information about project outcomes, utilizing the general press, magazines, radio, television, and web-based platforms. This will involve implementing targeted media campaigns aimed at raising public awareness and fostering understanding of the project's impact.

## 6. IMMUPARKNET: the roadmap for the future in PD

IMMUPARKNET is a pioneering international network that brings together experts, scientists, and clinicians from basic and clinical research with the aim of enhancing our understanding of the immune system’s role in PD. We believe this network will lead to significant advances in diagnosis and treatment for PD patients. During its implementation, IMMUPARKNET will establish a multidisciplinary ecosystem of experts in immunology and neurodegenerative diseases. This will ensure the continuous exchange of knowledge, and lead to the identification of common patterns and disease mechanisms, and the development of new therapeutic approaches. The network will be expanded progressively, and sustainability arrangements will be put in place. Funding opportunities will be pursued, including Horizon Europe, JPCOFUND 2 and European Partnerships. IMMUPARKNET aims to develop a new generation of researchers and physicians equipped with competencies derived from the cross-fertilization approach. This will be achieved through capacity-building measures such as Short-Term Scientific Missions (STSMs), yearly training schools, webinars, and an international PhD course. These scientists, at least 250 during the project and 1200 within two years of its end, will help translate IMMUPARKNET findings into new therapeutic approaches and tools. Additionally, IMMUPARKNET will provide new experimental and clinical data on PD pathogenesis, epidemiology, and prevention, opening new research pathways for therapeutic targets and resulting in a rich database of clinical and biological variables for future research. Moreover, uncovering the role of immunity in PD will lead to the development of valid models for the study of the immune component to potentially be used for translational research. This will also result in the discovery and validation of biomarkers in peripheral blood and other biological matrices, such as cerebrospinal fluid (CSF), to improve diagnosis, follow-up, and personalized therapy, including pharmaceuticals through drug-repurposing. In the medium term, biomarkers could include gene and protein expression in specific cells, extracellular vesicles, inflammatory and immune mediators, and immune cell and subset frequency. This is expected to lead to new opportunities for patent development and have a significant impact on related industry sectors. PD causes severe disabilities and dependence on others, with no treatments available to modify the disease's progression. IMMUPARKNET aims to address this unmet need by studying immunity in PD, which may also help to better understand the pathogenesis of non-motor symptoms, such as depression and anxiety. Treatments targeting immunity are being investigated in clinical and experimental neuropsychiatry, permitting this Action to act as a bridge between fields of research. PD not only affects patients but also their families, as they often become caregivers, which can negatively impact their well-being and productivity. Thus, improved scientific knowledge and therapeutics will positively affect a significant portion of the population, and alleviate the psychological and physical burden of PD. In addition, IMMUPARKNET's innovative therapeutic approaches are not only expected to modify the progression of Parkinson's disease by reducing the burden on individual patients and their families but lead to decreased healthcare costs thanks to a reduced need for interventions and an increased duration of life with limited or no disability. The direct and indirect costs related to Parkinson's disease are roughly equal in amount. By improving the physical condition of the patient, all indirect cost categories will significantly decrease, and there may be a 25–30% saving in total PD-related costs.

## Ethics and consent

Ethical approval and consent were not required.

## Data Availability

No data are associated with this article.
