# Supplementary material for: IMMUnity Unveiled: A Translational NETwork for tackling PARKinson's Disease – IMMUPARKNET
Source: Open Res Eur. 2025 Jan 23;4:119. Originally published 2024 Jun 19. [Version 2] doi: 10.12688/openreseurope.17547.2 (PMC11795026; doi:10.12688/openreseurope.17547.2)
Supplement: Peer review history

# Reviewer report: Reviewer response for version 2

**DOI:** 10.21956/openreseurope.20833.r50236

**Published:** 2025-02-04

**Authors:** Rifat Hamoudi

**Affiliations:** 1 University of Sharjah, Sharjah P.O. Box, United Arab Emirates; 2 Division of Surgery and Interventional Science, University College London, London, England, UK

I thank the authors for addressing the concerns. I have no further issues with and approve the article for indexing.

Where applicable, are recommendations and next steps explained clearly for others to follow? (Please consider whether others in the research community would be able to implement guidelines or recommendations and/or constructively engage in the debate)

Yes

Does the article adequately reference differing views and opinions?

Partly

Are all factual statements correct, and are statements and arguments made adequately supported by citations?

Yes

Is the rationale for the Open Letter provided in sufficient detail? (Please consider whether existing challenges in the field are outlined clearly and whether the purpose of the letter is explained)

Partly

Is the Open Letter written in accessible language? (Please consider whether all subject-specific terms, concepts and abbreviations are explained)

Yes

Reviewer Expertise:

Neurobiology, Immunology, Bioinformatics, and Pathology

I confirm that I have read this submission and believe that I have an appropriate level of expertise to confirm that it is of an acceptable scientific standard.

# Reviewer report: Reviewer response for version 2

**DOI:** 10.21956/openreseurope.20833.r50272

**Published:** 2025-01-25

**Authors:** Johannes CM Schlachetzki

**Affiliations:** 1 University of California San Diego, San Diego, California, USA

The present manuscript highlights the framework of IMMUPARKNET, a collaborative network of five working groups and 157 active members across 34 countries. It brings together expertise in studying the immune system's role in Parkinson’s disease. The main objectives are to facilitate work on developing consensus statements, technical protocols, guidelines, and novel therapeutic drugs, as well as outreach, dissemination, and training in this field of PD.

 The manuscript is very well written. In section 2, it would be helpful to highlight why the authors think studying the immune system in PD is important. The immune system responds to many pathologies and could be a bystander in the pathogenesis. Thus, for example, it would be helpful to emphasize further potential genetic evidence of immune system involvement and the possible interaction between synuclein and the adaptive immune system.

 It’s unclear how other researchers interested in this network could join.

Where applicable, are recommendations and next steps explained clearly for others to follow? (Please consider whether others in the research community would be able to implement guidelines or recommendations and/or constructively engage in the debate)

Yes

Does the article adequately reference differing views and opinions?

Yes

Are all factual statements correct, and are statements and arguments made adequately supported by citations?

Yes

Is the rationale for the Open Letter provided in sufficient detail? (Please consider whether existing challenges in the field are outlined clearly and whether the purpose of the letter is explained)

Yes

Is the Open Letter written in accessible language? (Please consider whether all subject-specific terms, concepts and abbreviations are explained)

Yes

Reviewer Expertise:

Immune system, neurodegenerative diseases including Alzheimer's disease and Parkinson's disease, Epigenetics

I confirm that I have read this submission and believe that I have an appropriate level of expertise to confirm that it is of an acceptable scientific standard.

# Reviewer report: Reviewer response for version 1

**DOI:** 10.21956/openreseurope.18962.r45118

**Published:** 2024-11-11

**Authors:** Rifat Hamoudi

**Affiliations:** 1 University of Sharjah, Sharjah P.O. Box, United Arab Emirates; 2 Division of Surgery and Interventional Science, University College London, London, England, UK

Gugu et al. provided timely open letter related to IMMUPARKNET which is a translational network for tackling Parkinson Disease (PD). 

 The open letter is a timely idea as it focuses on the immune response related to PD but the open letter has the following issues that needs to be addressed:

 1) It is best to add objectives to IMMUPARKNET as the mission stated is mainly related to logistics and policies and there is nothing scientific mentioned. So perhaps the authors can add section on just objectives in subsection of section 4. I suggest the authors make the objectives scientific related to PD

 2) The authors need to briefly discuss PD patient recruitment or monitoring. The authors are from different countries in Europe so some mechanism of patients recruitment and inclusion/exclusion criteria needs to be mentioned

 3) The article should benchmark this group against other PD groups worldwide and how this group can complement other similar groups on PD translational work

Where applicable, are recommendations and next steps explained clearly for others to follow? (Please consider whether others in the research community would be able to implement guidelines or recommendations and/or constructively engage in the debate)

Yes

Does the article adequately reference differing views and opinions?

Partly

Are all factual statements correct, and are statements and arguments made adequately supported by citations?

Yes

Is the rationale for the Open Letter provided in sufficient detail? (Please consider whether existing challenges in the field are outlined clearly and whether the purpose of the letter is explained)

Partly

Is the Open Letter written in accessible language? (Please consider whether all subject-specific terms, concepts and abbreviations are explained)

Yes

Reviewer Expertise:

Neurobiology, Immunology, Bioinformatics, and Pathology

I confirm that I have read this submission and believe that I have an appropriate level of expertise to confirm that it is of an acceptable scientific standard, however I have significant reservations, as outlined above.

# Response

**Published:** 2024-12-20

**Authors:** Inês Figueira

**Affiliations:** Not Applicable, Portugal

We kindly acknowledge the reviewer’s comments regarding Gugu et a. IMMUPARKNET COST Action Open Letter on Open Research Europe. Below, we clarify the aspects which have been appointed by the reviewer and we also upload a revised version of the manuscript in order to address all the concerns raised. We believe that with all these edits, the overall quality of the document has dramatically increased, and we appreciate very much once more the extremely valid inputs received.  Regarding the first aspect raised in relation to the lack of scientific objectives being highlighted in the manuscript (Point 1), we kindly appreciate the comments by the reviewer, and we have slightly modified the text to make it more clear. In fact, a COST Action like IMMUPARKNET is not mainly centered into delve scientific breakthroughs but mainly on increasing network/collaboration opportunities and raising awareness about a particular topic that is still underlooked (in our case, the importance of studying immune response in the context of PD). Nevertheless, several scientific objectives are envisaged, such as the publication of review manuscripts and opinion articles (published and in preparation), the participation in dissemination activities (talks, outreach events) and even contacts with potential stakeholders for the future sustainability plan of the action. Therefore, all these more scientifically related outputs have been presented in more detail in the revised version of the paper.  Regarding Point 2, concerning patient recruitment or monitoring hurdles among different European countries (and even between different institutions), we fully agree with the reviewer’s comment and we have added discussion related to the topic in the revised manuscript. In fact, not only that but clear guidelines to educate clinicians on how to address immune response studies in clinical practice with PD patients are missing, a knowledge gap that our Action has united efforts to fill out through peer-review publications, currently submitted (Lill, C., di Flora, A., Sechi, L., Pereira, F., Moisoi, N., Nikolovski, N., Aktekin, M., Gursoy-Ozdemir, Y., Deecke, L., Cossu, D., Homann, J., Simula, E., Keskinöz, E. N., Öz-Arslan, D., Bayar Muluk, N., Gonçalves, M., Pirovano, E., Comi, C., & Marino, F. (Dys)regulation of the immune system in Parkinson’s disease: Methodologies, techniques, and current knowledge. Aging and disease (under revision); Laura Muñoz-Delgado, Caroline H. Williams-Gray, Gaetan Garraux, Diego Clemente, Özgür Öztop Çakmak, Julia C. Greenland, Inês Figueira, Isabel Machín-Díaz, Elenamaria Pirovano, Alessia di Flora, Clotilde Tournerie, Stamatia Papoutsopoulou, Sibel Ertan, Pablo Mir, Cristoforo Comi. Recommendations for Clinical Observational Studies of the Immune System in Parkinson’s disease. npj Parkinson's Disease (submited))    Regarding the third aspect raised, we have added in the manuscript as well a brief overview concerning the framing of IMMUPARKNET COST Action in comparison to other PD-related initiatives worldwide. As a COST Action initiative, IMMUPARKNET goal is not in any case to compete or even replace other European and/or Global initiatives, such as Parkinson Europe or even the Parkinson’s Foundation or World Parkinson Coalition. Our mission is to educate researchers, clinicians, and even patients and caregivers on the importance of immune response and immune symptom management (especially at the early stages) for better performance and quality of life. Through this COST Action, we are working as a driving force to promote knowledge increase in the field and, in the near future, we hope to mirror the results from the synergisms that have been established between members and outputs gathered (joint grant applications, cohorts, shared samples, peer-reviewed publications, conferences, etc.) as a solid proof of the importance of this consortium.
